# Establishment of a Novel In Vitro and In Vivo Model to Understand Molecular Carcinogenesis of Endometriosis-Related Ovarian Neoplasms

**DOI:** 10.3390/ijms26051995

**Published:** 2025-02-25

**Authors:** Hasibul Islam Sohel, Tohru Kiyono, Umme Farzana Zahan, Sultana Razia, Masako Ishikawa, Hitomi Yamashita, Kosuke Kanno, Shahataj Begum Sonia, Kentaro Nakayama, Satoru Kyo

**Affiliations:** 1 Department of Obstetrics and Gynecology, Faculty of Medicine, Shimane University, Izumo 693-8501, Japan; hasibulsohel1167@gmail.com (H.I.S.); farzanashormi99@gmail.com (U.F.Z.); m-ishi@med.shimane-u.ac.jp (M.I.); meme1103@med.shimane-u.ac.jp (H.Y.); kanno39@med.shimane-u.ac.jp (K.K.); sbsonia1995@gmail.com (S.B.S.); 2 Exploratory Oncology Research and Clinical Trial Center (EPOC), National Cancer Center, Kashiwa 277-8577, Japan; tkiyono@east.ncc.go.jp; 3 Department of Legal Medicine, Shimane University Faculty of Medicine, 89-1 Enya-Cho, Izumo 693-8501, Japan; raeedahmed@yahoo.com; 4 Department of Obstetrics and Gynecology, East Medical Center, Nagoya City University, Nagoya 464-8547, Japan

**Keywords:** endometriosis, carcinogenesis, ERONs, clear cell carcinoma, endometrioid carcinoma, tumor immune microenvironment

## Abstract

The molecular mechanisms through which endometriosis-related ovarian neoplasms (ERONs) develop from benign endometrioma remain unclear. It is especially a long-standing mystery why ovarian endometrioma has the potential to develop into two representative histological subtypes: endometrioid ovarian carcinoma or clear cell ovarian carcinoma. This study aimed to investigate the molecular carcinogenesis of ERONs using newly developed in vitro and in vivo carcinogenesis models. Epithelial cells were isolated and purified from surgically removed benign endometrioma samples, followed by immortalization by overexpressing *cyclin*D1/*CDK4* in combination with the human *TERT* gene. Immortalized cells were subjected to various molecular manipulations by combining knockout or overexpression of several candidate drivers, including *ARID1A*, *KRAS*, *PIK3CA*, *AKT*, and *MYC*, based on previous comprehensive genome-wide studies of ERONs. These cells were then inoculated into immunocompromised mice and evaluated for malignant transformation. Inoculated cells harboring a combination of three genetic alterations successfully developed tumors with malignant features in mice, whereas those with two genetic manipulations failed to do so. Especially, *ARID1A* gene knockout, combined with overexpressing the *KRAS* oncogenic mutant allele (or overexpressing AKT) and c-Myc overexpression led to efficient tumor formation. Of note, these three combinations of genetic alterations produced tumors that histologically represented typical clear cell carcinoma in SCID mice, while the same combination led to tumors with endometrioid histology in nude mice. A combination of *ARID1A* mutation, *KRAS* mutation or AKT activation, and c-Myc overexpression were confirmed to be the main candidate drivers for the development of ERONs, as suggested by comprehensive genetic analyses of ERONs. A tumor immune microenvironment involving B-cell signaling may contribute to the diverse histological phenotypes. The present model may help to clarify the molecular mechanisms of ERON carcinogenesis and understand their histological diversity and novel molecular targets.

## 1. Introduction

Endometriosis is an estrogen-dependent condition that affects 7–15% of women of reproductive age with pain, infertility, and so on [1,2]. Clinicopathological, genetic, and epidemiological investigations have demonstrated an increased risk of epithelial ovarian cancer, especially ovarian clear cell carcinoma (OCCC) or ovarian endometrioid carcinoma (OEC), which are known as endometriosis-related ovarian neoplasms (ERONs) [3,4] and are the most severe consequences of endometriosis [5]. Historically, morphologic investigations have repeatedly shown a link between OCCC or OEC and endometriosis, and it is now widely accepted that the majority of these tumors develop from benign endometriotic cysts [4,6]. In Japan, it is known that women with ovarian endometriotic cysts have a significantly higher risk of developing ovarian cancer, with a standardized rate proportion of 8.95. Among these cancers, 39% were OCCC and 35% were OEC [7].

Clinically, endometriosis, particularly endometriotic cysts, should not be generally treated as precursors for ERONs, but those in older patients or of larger size have an increased risk for developing ERONs. ERONs fall under the category of type I carcinogenic pathway of ovarian carcinomas, usually originating from benign precursor lesions, with their molecular genetic characteristics extensively studied [8] and mutations of the *ARID1A* gene are most potentially associated with ERONs. Many studies indicate that *ARID1A* mutations occur in approximately 95% of OCCC and 30% of OEC, suggesting their role as an early molecular event in the progression of ERONs [4,9,10,11,12,13,14,15]. Each of the ERON subtypes has also been found to have a number of common mutations, including *PIK3CA* (15.8%), *PPP2R1A* (7%), *P53* (5%), *PTEN* (37%), *KRAS* (11.1%), *ZNF21* (20%), c-Myc (20%), as well as AKT (9%) [16]. This evidence suggests that these frequent mutations are responsible for the development of ERONs.

Early detection of OCCC is usually associated with a good prognosis. However, in advanced stages, this cancer type responds poorly to initial platinum-based chemotherapy. As a result, the prognosis is generally worse than serous or endometrioid adenocarcinomas diagnosed at the same stage [17,18,19,20]. OEC often has a good prognosis, but some patients face challenges such as drug resistance, recurrence, or even death [21]. Despite the above comprehensive genetic analyses, the precise molecular processes by which ovarian endometriosis leads to OCCC and OEC are not yet fully understood. Especially, the number of genetic/epigenetic alterations or the minimal requirements for the development of ERONS as well as the specific combinations for efficient carcinogenesis remain unclear.

To clarify these questions, we established an in vitro stepwise carcinogenesis model using immortalized endometriotic epithelial cells, in which various genetic alterations were introduced in specific combinations, and tumorigenicity was evaluated with immunocompromised mice.

## 2. Results

### 2.1. Immortalization of Primary Endometriotic Epithelial Cells

**Figure 1:**
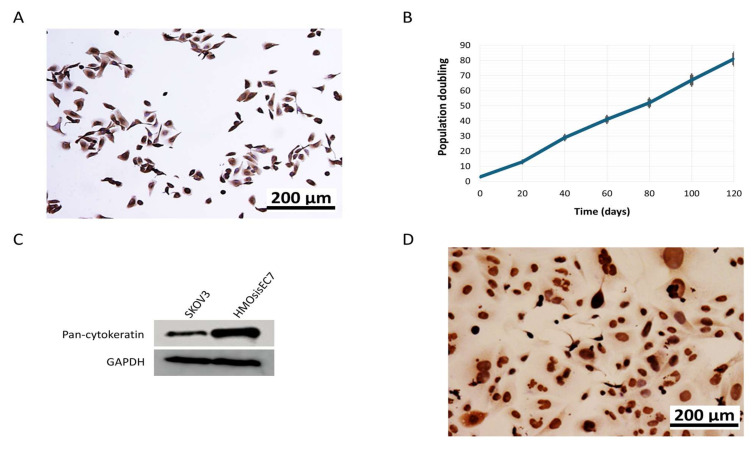
Characterization of immortalized HMOsisEC7 cells. (**A**) Immunocytochemistry of HMOsisE7 cells with pan-cytokeratin confirming epithelial morphology. (**B**) Growth curve of HMOsisE7 cells. Population doublings are shown at each time point (days after culture). (**C**) Western blot analysis for pan-cytokeratin. SKOV3 cells are used as a positive control. (**D**) PAX8 expression indicates the absence of stromal cell contamination. The full-length Western blot gel image is available in Supplementary Figure S4. GAPDH, glyceraldehyde3-phosphate dehydrogenase.

Endometriotic tissues were collected from a 53-year-old patient with ovarian endometriosis. The tissues were gently digested using collagenase type-III and glandular cells were isolated by microscopic manipulation to avoid the contamination of stromal cells as previously described [22,23]. Then, the cells were subjected to primary culture at 37 °C in a 5% CO_2_ incubator. The cells were confirmed to display typical epithelial morphology (Figure 1A). It is well known that two obstacles (primary senescence and telomere-dependent senescence) hinder the long-term in vitro survival of epithelial cells. To address this issue, we aimed to immortalize the cells for further experiments by overexpressing the p16-insensitive mutant of CDK4 (CDK4R24C), cyclin D1, and TERT by lentiviral transfection to disrupt the Rb pathway and activate telomerase, respectively, according to previous studies [24,25,26]. After transfection, the cells continued to increase by more than 90 population doublings, and the morphological features of epithelial cells were maintained (Figure 1B), demonstrating that the cells, named HMO-sisEC7, were immortalized. Western blotting and immunocytochemistry confirmed significant expression of pan-cytokeratin (Figure 1C), a representative marker of epithelial cells. Additionally, we confirmed significant PAX8 expression (Figure 1D), which may exclude the possibility of stromal cell contamination. These immortalized cells failed to form colonies in soft agar and tumors in nude mice, even nine months after engraftment confirming that HMOsisEC7 did not acquire a transformed phenotype.

Along with the immortalization process, we conducted whole-exome profiling to identify any pre-existing genetic mutations in the ovarian endometrioma samples used to create HMOsisEC7 cells. No significant genetic mutations, insertions/deletions, or copy number alterations of known oncogenes or tumor suppressor genes were observed (Supplementary Figure S1 and Supplementary Table S1), indicating that HMO-sisEC7 cells were successfully immortalized from benign endometrioma cells without any of the cancer-associated genetic alterations.

### 2.2. Genetic Manipulation of Immortalized Endometriotic Epithelial Cells

**Figure 2:**
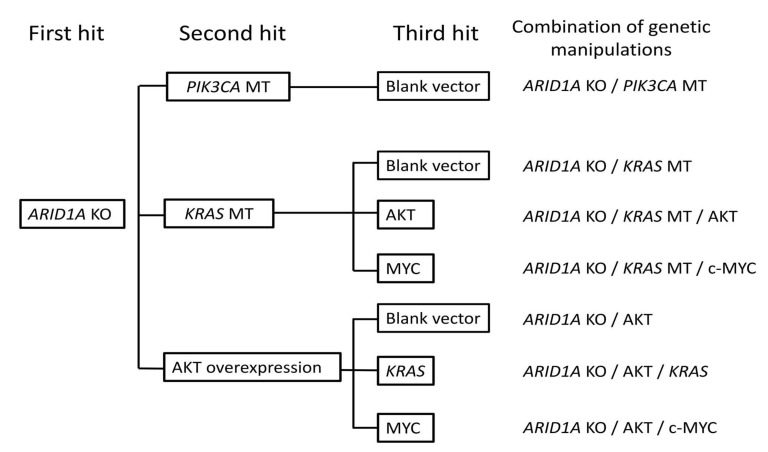
Genetic manipulations introduced into immortalized HMOsisEC7 cells. The order of the introduction of genetic mutation/overexpression is shown, with the combination of manipulations indicated. KO, knockout. MT, mutation.

According to The Cancer Genome Atlas (TCGA), frequent somatic mutations detected in ERONs include *ARID1A* (95%), *PIK3CA* (15.8%), *KRAS* (11.1%), AKT (9%), and c-Myc (20%), while other types of mutations are less common [15,16], and the loss of *ARID1A* is especially notable [27,28,29]. Based on these findings, we hypothesized that the loss of *ARID1A* is an initiating event indispensable for the carcinogenesis of ERONs. Therefore, we attempted the knockout of *ARID1A* as the first hit in HMOsisEC7 cells using the CRISPR-Cas9 system (Supplementary Figure S2). Subsequently, we sought to introduce additional oncogenic mutations. The second hit selected was RAS/ERK or PI3K/AKT pathway activation, in which we overexpressed mutant *KRAS* or *PIK3CA* alleles or constitutively activated AKT alleles [22,23,30]. Furthermore, the c-Myc (c-MycT58A) and AKT (mouse Akt1 fused with a myristoylation signal) were overexpressed as the third hit [23] (Figure 2).

**Figure 3:**
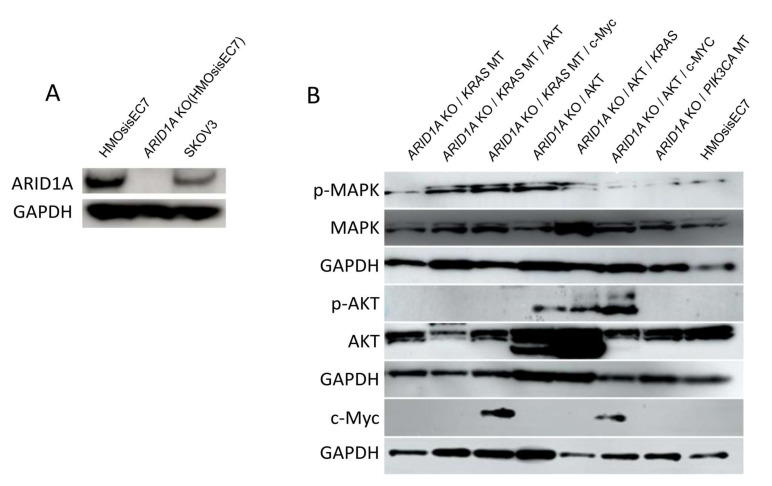
Western blot analysis confirms the expression of ARID1A or other genes in HMOsisEC7 cells with various genetic manipulations. (**A**) Expression of *ARID1A* in cells with *ARID1A* KO in comparison with parental HMOsisEC7 cells and the SKOV3 cell line. (**B**) Expression of various target genes in cells with overexpressing *KRAS* or *PIK3CA* mutant alleles or overexpressing constitutively activated AKT or c-Myc. The name of each cell type and the various genetic manipulations are listed above the gel images. The full-length Western blot gel image is available in Supplementary Figure S5. KO, knockout. MT, mutation; OCCC, ovarian clear cell carcinoma. OEC, ovarian endometrioid carcinoma, GAPDH, glyceraldehyde3-phosphate dehydrogenase.

The efficiency of knockout or overexpression was confirmed by Western blot analysis (Figure 3). The morphology was not altered by these genetic manipulations compared to the parental HMOsisEC7 cells (Supplementary Figure S3).

### 2.3. The Effects of Genetic Alterations on the Growth Property of Immortalized Endometriotic Epithelial Cells

**Figure 4:**
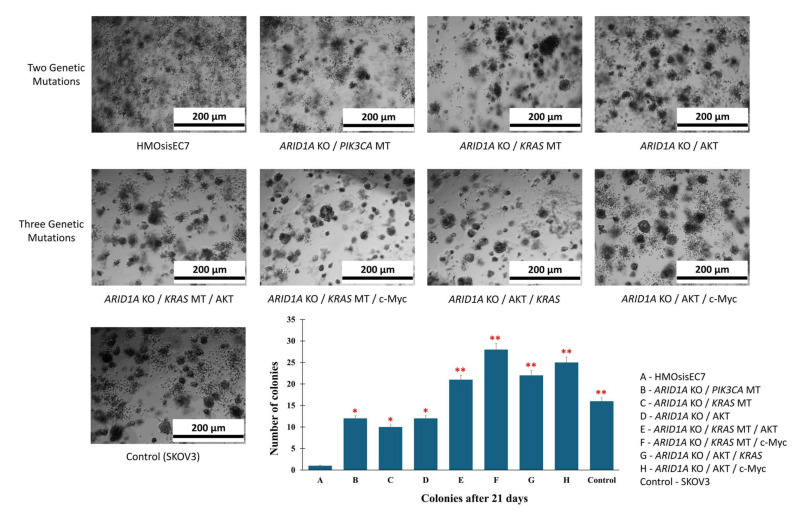
Anchorage-independent growth assay of HMOsisEC7 cells with various genetic manipulations. Photographs of representative colonies with various genetic manipulations by transmitted light microscopy. The corresponding quantification is shown in the bar graph, representing the number of colonies (>50 µm) after 21 days of seeding. * *p* < 0.05, ** *p* < 0.01. KO, knockout. MT, mutation; OCCC, ovarian clear cell carcinoma. OEC, ovarian endometrioid carcinoma.

We next evaluated the effects of genetic alterations on the growth property of cells. MTT assays revealed that HMOsisEC7 cells with three genetic modifications exhibited accelerated growth in vitro. Similarly, colony-forming assays showed that cells with three genetic modifications had enhanced anchorage-independent growth, forming larger and more numerous colonies compared to cells with two genetic modifications or parental cells (Figure 4). Furthermore, invasion assays and wound healing assays clearly showed that HMOsisEC7 cells with *ARID1A* KO, *KRAS* MT or overexpressing AKT, and overexpressing c-Myc exhibited the highest proliferative, invasion, and wound healing ability compared to parental or other mutational combinations (Supplementary Figure S4A–C).

### 2.4. The Effects of Genetic Alterations on the Tumor Forming Ability in Mice

HMOsisEC7 cells with *ARID1A* KO and additional genetic manipulations were subcutaneously transplanted into immunodeficient female C.B-17/Icr SCID and BALB/c nude mice. We observed that only the triple-mutant HMOsisEC7 cells developed macroscopic tumors, while the double-mutant cells did not, even at a high cell count of 1 × 10^7^ (Table 1 and Table 2).

**Figure 5:**
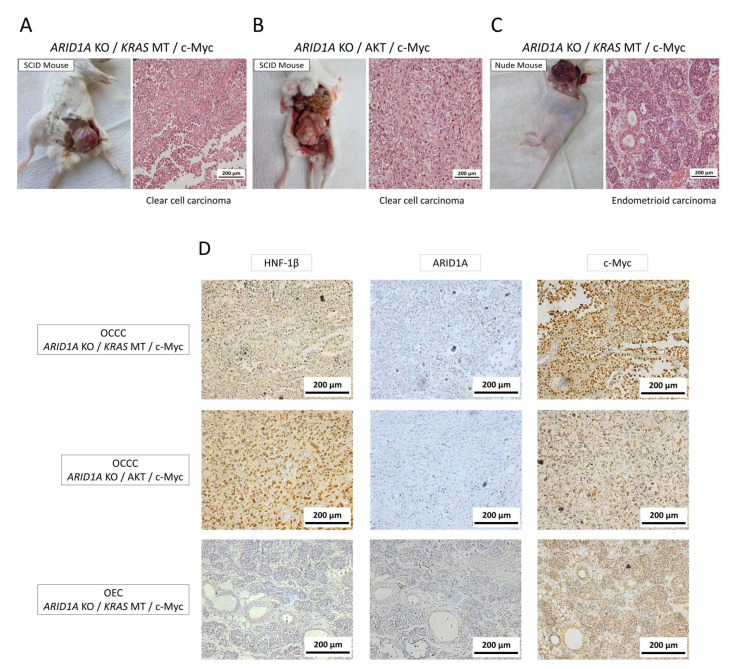
Tumor formation assay in immunocompromised mice. HMOsisEC7 cells with three genetic manipulations, *ARID1A* KO, *KRAS* MT, and c-Myc overexpression (**A**) or *ARID1A* KO, AKT, and c-Myc overexpression (**B**) can form tumors in SCID mice, representing histological features of OCCC with significant HNF1-β expression. In contrast, HMOsisEC7 cells with three genetic manipulations, *ARID1A* KO, *KRAS* MT, and c-Myc overexpression (**C**) can form tumors in nude mice, showing histological features of OEC lacking HNF-1β expression. The lack of ARID1A expression and overexpression of c-Myc in these mouse tumors were confirmed by IHC (**D**). KO, knockout. MT, mutation; OCCC, ovarian clear cell carcinoma. OEC, ovarian endometrioid carcinoma.

Histological examination of the xenograft tumors revealed distinct features associated with the mouse species. SCID mice inoculated with HMOsisEC7 cells with *ARID1A* KO, *KRAS* MT, and overexpressing c-Myc (4/5) or those with *ARID1A* KO, overexpressing AKT and overexpressing c-Myc (3/5) developed phenotypically malignant tumors displaying solid sheets and targetoid patterns characteristic of OCCC (Figure 5A,B). Immunohistochemical and immunocytochemical analyses revealed the apparent nuclear expression of HNF-1β. Interestingly, when HMOsisEC7 cells with *ARID1A* KO, *KRAS* MT, and overexpressing c-Myc were inoculated into nude mice, macroscopic tumors developed that were morphologically typical of OEC with abundant luminal mucin (2/5) (Figure 5C). All of these tumors formed slowly within 8 to 10 months after inoculation, probably reflecting non-aggressive clinical features of ERONs, quite different from the high-grade serous carcinoma model we established from fallopian tube cells [23]. An IHC study of these mouse tumors confirmed the lack of significant expression of ARID1A, as well as overexpression of c-Myc (Figure 5D), and also Western blot analysis, confirmed the activation of both the RAS/ERK or PIK3CA/AKT signaling pathways and epithelial origin (Supplementary Figure S5). These findings indicated that the lack of *ARID1A*, *KRAS* MT (or AKT overexpression), and c-Myc overexpression are the driver candidates essential for the development of ERONs.

## 3. Discussion

In this study, we aimed to establish an in vitro and in vivo model to understand driver mutations implicated in the development of ERONs.

A typical endometriosis is the putative precursor of ERONs, in which *ARID1A* mutations are already observed. This is quite different from other histological ovarian cancers such as high-grade serous ovarian carcinomas, in which an *ARID1A* mutation is relatively rare while *p53* mutations are very frequent as essential drivers [3,11,12,31,32,33]. These findings imply that *ARID1A* mutation is a unique and indispensable driving factor in the development of ERONs [12,34]. Furthermore, our recent investigations, along with data from TCGA, have elucidated that *PIK3CA* MT, *KRAS* MT, AKT activation, and c-Myc amplification are especially frequent in ERONs and, therefore, may be involved in the carcinogenesis [15,16]. This evidence is the rationale for our focus on particular mutations introduced into our in vitro stepwise model.

The in vitro stepwise carcinogenesis model was established from primary cultured endometriotic epithelial cells isolated from surgically removed ovarian endometrioma samples, followed by immortalization through the overexpression of *cyclin D1*, *CDK*4, and *hTERT* genes [23,24,25,26,35]. We successfully created immortalized HMOsisEC7 cells retaining phenotypic and immunohistochemical features of primary cultured endometriotic epithelial cells. The whole-exome sequencing of the original endometrioma samples revealed that the tumor did not have representative cancer-associated mutations, indicating that HMOsisEC7 cells may be ideal for the use as the in vitro stepwise model.

We first knocked out the *ARID1A* gene in HMOsisEC7 cells, then additionally introduced *KRAS* mutations and/or overexpressed AKT separately and/or overexpressed c-Myc, generating four types of three genetic modifications (*ARID1A* KO, *KRAS* MT, and overexpressed AKT; *ARID1A* KO, *KRAS* MT, and overexpressed c-Myc; *ARID1A* KO, overexpressed AKT and *KRAS* MT; *ARID1A* KO, overexpressed AKT, and overexpressed c-Myc), which showed higher proliferation, invasion, migration, and colony formation abilities compared to cells with two genetic changes or the parental HMOsisEC7 cells. Furthermore, our mouse tumorigenicity assays revealed that only the cells with three genetic combinations (*ARID1A* KO, *KRAS* MT (or AKT overexpression), and c-Myc overexpression) successfully formed malignant tumors, suggesting that they are driver candidates in the development of ERONs, which aligns with previous studies in which the establishment of three genetic alterations showed the potential for tumorigenesis [36,37]. These combinations of drivers are unique in the development of other ovarian cancer types, especially the obvious difference from those of high-grade serous carcinoma, with an absolute requirement for *p53* mutations. Based on genome-wide studies such as TCGA [16], most ERON cases lack *p53* mutations and, therefore, do not clinically represent aggressive behavior. This study required a long-term period to confirm sufficient tumor growth in mice with this specific combination of genetic alterations, demonstrating that such results of our in vitro carcinogenesis model precisely reflect the growth features of ERONs.

Of particular interest is the histological diversity in tumors formed by the same genetic arrangements of *ARID1A*, *KRAS* MT, and c-Myc overexpression, depending on the mouse species, SCID or nude mice; the former represents OCCC, and the latter demonstrates apparent OEC. There have been some controversies about the molecular mechanisms of diverse histological phenotypes of ERONs, especially OCCC and OEC phenotypes, in light of their similar genetic abnormalities. Cochrane et al. demonstrated that different histological phenotypes could arise from different cells of origin; OCCC expressed the ciliated cell marker cystathionine γ-lyase (CTH) while OEC expressed the secretory cell marker methylenetetrahydrofolate dehydrogenase 1 (MTHFD1) [38], underscoring that OCCC and OEC arise from distinct cell types. However, Kolin et al. highlighted the limitations of this model, in that marker expression alone cannot identify the cells of origin due to phenotypic plasticity. They also suggested that lineage tracing or transplantation assays are needed for accurate identification. It is also noted that the stem cells in the female genital tract impact tumor differentiation, questioning whether there are multiple stem cell niches or a single stem cell population. They hypothesized that the different histological subtypes of tumors that develop from a single precursor lesion, such as endometriosis, may result from the interplay between the originating cell and the influence of genetic and epigenetic factors, along with the tumor microenvironment [39].

Recently, Beddows and colleagues demonstrated that the cell state rather than cell type gives rise to different histological subtypes; OCCC expressed HNF-1β and represented genetic features of secretory phase endometrial cell lineages while OEC showed those of proliferative phase endometrial cell lineages, highly expressing estrogen receptor 1 (ERS1). Especially, *ESR1* gene expression was significantly inhibited by DNA methylation in OCCC, while cellular iron retention signaling was much enhanced in this tumor type [40].

Our data demonstrated that tumor immune microenvironments (TIMEs) may affect phenotypic features of ERONs, because SCID mice lack both T- and B-cells while nude mice lack only T-cells, meaning that the presence of B-cells may be at least partly involved in histological diversity. The tumor microenvironment consists of the surrounding cellular environment, including blood vessels, immune cells, other non-tumor cells, the extracellular matrix, and signaling molecules. These elements interact with each other and with tumor cells, affecting tumor growth and behavior. Key processes like tumor cell proliferation, invasion, epithelial–mesenchymal transition, angiogenesis, and drug resistance are influenced by these interactions, particularly within the TIMEs [41]. Thus, TIMEs have a role in tumor management and could offer novel treatment approaches. Fridman et al. stated that until recently, the importance of B-lymphocytes in this regard was undervalued. B-cells can have two opposing effects; they can either promote chronic inflammation, angiogenesis, or immunosuppression by the creation of immune complexes or complement activation, or they can increase T-cell responses and destroy tumor cells through ADCC (antibody-dependent cellular cytotoxicity). However, the understanding of the heterogeneity and diversity of B-cell subsets in tumors remains inadequate, which may pose major challenges for targeting B-cells in oncological therapy. Additionally, a significant number of intra-tumoral B-cells may merely act as bystanders rather than as antitumoral, much like many T-cells [42]. If the TIMEs, especially involving B-cell pathways, are responsible for different histological subtypes, B-cells or associated signaling may serve as both a prognostic marker and a potential therapeutic target. However, further studies are necessary to fully elucidate this issue, as different histological subtypes arise in different animals with varying immunity due to differences in the tumor immune microenvironment (TIME).

Although we have successfully established an in vitro and in vivo carcinogenesis model of human ERONs, two major limitations remain. First, drug sensitivity has not been evaluated, which is essential for assessing therapeutic responses. Second, a comprehensive analysis of the tumor immune microenvironment is necessary to understand the factors driving histological diversity. Addressing these aspects will be crucial for future studies.

## 4. Materials and Methods

### 4.1. Isolation and Primary Culture of Human Endometriotic Epithelial Cells

The endometriotic tissue samples were collected from surgically removed ovarian endometriomas of a 53-year-old patient at Shimane University Hospital. The patient provided written informed consent for her clinical and pathological tissue specimens to be used in this study. Approval of this study was obtained from the ethics review board of Shimane Medical University (IRB No. 20070305-1 and 20070305-2). Following collagenase digestion under sterile conditions, endometriotic epithelial cells were isolated and purified using the method described in our previous study. These cells were subjected to primary culture as previously described [22].

### 4.2. Whole-Exome Sequencing of Ovarian Endometriotic Tissues

The endometriotic tissue samples were post-operatively subjected to hematoxylin-eosin (HE) staining for pathological diagnosis. The DNA of endometriotic lesions was macroscopically collected and extracted from HE sections and subsequently subjected to whole-exome sequencing to check for preexisting genetic alterations; the techniques utilized for genome sequencing have been previously discussed [23]. The Agilent 2000 Tape Station (Agilent Technologies, Santa Clara, CA, USA) was first used to assess DNA integrity. Subsequently, Illumina MiSeq (Illumina, San Diego, CA, USA) whole-exome sequencing using enriched amplicons was performed. The sequencing data were analyzed using the Genome Jack bioinformatics pipeline (Mitsubishi Space Software Corp., Tokyo, Japan). High analytical sensitivity and specificity were ensured throughout the investigation by using sequence alignment, variant calling, variant filtering, variant annotation, and variant prioritizing.

### 4.3. Immortalization of Endometriotic Epithelial Cells

We incorporated and overexpressed cDNAs (complementary DNAs) encoding hu man TERT (telomerase reverse transcriptase), cyclin D1, and CDK4^R24C^ into primary cultured endometriotic epithelial cells by lentiviral transfer as previously described [22,23]. Thereafter, the population doubling (PD) of cultured cells was assessed, and immunohistochemical analyses were performed to assess the expression of several markers. Finally, the immortalized cells were named HMOsisEC7 cells.

### 4.4. Genetic Manipulations of Immortalized HMOsisEC7 Cells

Using CRISPR-Cas9, we sought knockout (KO) of the *ARID1A* gene using the technique described in our previous study [43]. Briefly, immortalized HMOsisEC7 cells at passage 18 were transfected with piggyback vectors, PB-TAC-ERN-3xFlag-hCas9, PB-TKbsd-U6/H1R-ARID1A-gRNA401-394, PB-TKbsd-U6/H1R-ARID1A-gRNA416-423, and pCAG-PBase-M282V as well as 0.1 mg of pCMV-EGFP in 100 mL of OptiMEM medium and pulsed using a NEPA21 (Nepagene Co., Ltd., Ichikawa City, Chiba, Japan). Cells were cultivated in the presence of 8 mg/mL of Blasticidin S, 100 mg/mL of G418 for 7 days, and then treated with 1 mg/mL of doxycycline for two weeks. These 24-well isolated colonies were trypsinized and propagated in a 24-well plate. Genomic DNA was extracted from each clone and amplified by Western blot and PCR using the forward primer 5’-gatcagatgggcaagatgagac-3’ and the reverse primer 5’-gtacctgtgtgaccagggagtaagtagt-3’ to confirm *ARID1A* status; a specific clone with a homologous 86 bp deletion between nt 1185 and 1270 of the *ARID1A* coding sequence was further propagated, multiplied, and used in additional studies.

Additionally, the cDNAs of the oncogenic mutant *KRAS* (KRAS^G12V^), *PIK3CA* (PIK3CA^E545K^), an active form of AKT (myristylation signal-fused human Akt1, provided by Dr. Goto from Aichi Cancer Research Institute, Nagoya, Japan), as well as an amplified form of AKT (myristylation signal-fused mouse Akt1, also provided by Dr. Goto) and *MYC* (MYC^T58A^) were cloned into retroviral vector plasmids (pCMSCVPpuro-KRASG12V, PB-TAC-ERN-3xFLAG-PIK3CAE545K, pCLMSCVpuro-myr-hAKT1-Myc, pCMSCV-EM7-bsd-myr-mAkt1 and pCMSCV-EM7-bsd-MYCT58A, respectively) through recombination using the Gateway system (Invitrogen, Carlsbad, CA, USA). Then, lentiviral infection was conducted using human mutant *KRAS*, *PIK3CA*, and active myr-AKT expression vectors to establish cells overexpressing these mutant oncogenes, followed by constitutively overexpressing AKT and c-Myc through site-directed mutagenesis. The resulting cell lines and the specific combinations of introduced mutations are summarized in Figure 2. These cell lines were cultured in F-medium supplemented with 10 µM Y-27632 [44] 500 nM DMH-1, 500 nM A-83-01, 17β-estradiol, 100 uM/mL penicillin, and 100 mg/mL and maintained in an incubator with 5% CO_2_ at 37 °C.

### 4.5. Analysis of Population Doubling

Cells were initially seeded at a density of 1 × 10^5^ cells/mL in a 25-cm^2^ dish. When they covered 80% of the dish’s surface, they were passaged. The population doubling level (PDL) was determined using the formula: PDL = log2(a/b), where “a” is the final cell count after passage and “b” is the initial number of seeded cells [45].

### 4.6. Immunohistochemical Analysis

HMOsisEC7 cells were placed on Lab-Tek chamber slides (Thermo Fisher Scientific, Waltham, MA, USA) for 24 h. The cells were permeabilized with 0.1% Triton X-100, then fixed with 4% formalin before being treated with primary antibodies against pan-cytokeratin and PAX8 proteins overnight at 4 °C (Supplementary Table S2). Next, PBS washes were performed. The cells were then treated with the secondary antibody for 1 h at room temperature, and a Histofine SAB-PO kit (Nichirei, Tokyo, Japan) was used to detect the presence of the secondary antibody.

Mouse xenograft tumor immunohistochemistry (IHC) was carried out on paraffin-embedded tissues. Briefly, successive 5-µm-thick sections of paraffin-embedded tissues were cut. A select few slides were used for IHC, and others were stained with HE for histological analysis. Deparaffinized sections were used for IHC, and they were treated with HNF-1β, ARID1A, and c-Myc proteins overnight at 4 °C (Supplementary Table S2). Tris-EDTA buffer (pH 9, Ref-S3467, Dako, Carpinteria, CA, USA) was used for antigen retrieval. Using a light microscope, samples were evaluated by a pathologist blinded to the clinicopathological variables.

### 4.7. Western Blot Analysis

Cell pellets were lysed in Laemmli sample buffer (Bio-Rad, Hercules, CA, USA) with 5% beta-mercaptoethanol (Sigma-Aldrich Japan, Tokyo, Japan). LDS buffer and sample-reducing buffer were then added to the protein sample and heated for 5 min at 101 °C, and the sample was kept on ice for 1 min. Then, the sample was centrifuged for 5 min at 15,000 rpm. Furthermore, 10 µL of protein marker and 18 µL of protein sample were loaded into sodium dodecyl sulfate (SDS) polyacrylamide gel electrophoresis (Invitrogen) and transferred to polyvinylidene fluoride membranes using Bio-Rad semi-dry trans-blotters (Trans-Blot^®^ SD cell, Bio-Rad Laboratories, Hercules, CA, USA). The membranes were then blocked in LI-COR blocking buffer (LI-COR, Lincoln, NE, USA) for 1 h at room temperature (25 °C). Primary antibodies (Supplementary Table S2) were then added, and the membranes were incubated overnight at 4 °C on a shaker. The membranes were washed four times for 5 min each with TBST and treated with secondary antibodies (goat anti-mouse or goat anti-rabbit IR-Dye 670- or 800 CW labeled) for 1 h at room temperature. The probed membranes were then washed with TBST and imaged using an LI-COR Odyssey scanner (LI-COR, Lincoln, NE, USA). Odyssey 3.0 analytical software (Model-9120, S/N: ODY-2280, LI-COR) was used to determine the raw intensity and near-infrared fluorescence values; intra-lane background signals were eliminated, and boxes were manually regulated over each band of interest.

### 4.8. Cell Viability Assay

The MTT assay was used to measure the proliferation of HMOsisEC7 cells in a 0.5% growth media [46]. The cells were seeded in 96-well plates at a density of 4000 cells per well, followed by the MTT assay. The results are presented as mean ± standard deviation (SD), based on data acquired from studies performed in triplicate.

### 4.9. The Scratch-Wound Healing Assay

Cells (1 × 10^6^) were sown in 6-well plates and cultivated until confluence. To generate an acellular region, the cell surface was scraped with a 200 µL pipette tip, then gently washed with culture fluid twice to remove floating cells. The rate of fault closure was observed for 48 h.

### 4.10. In Vitro Matrigel Invasion Assay

Cell invasion was measured using chambers with 8 µm holes (Discovery Labware, Inc., Woburn, MA, USA). Each lower chamber contained 900 µL of DMEM with 20% FBS, while the top chamber was seeded with 25,000 cells suspended in 350 µL of serum-free media. The Matrigel was gently removed with a cotton swab after 24 h of culture. The membranes were fixed with 4% paraformaldehyde and stained with Giemsa. The total quantity of migratory cells was counted in five non-overlapping areas using a light microscope.

### 4.11. The Clonogenic Assay

Cells were sown at a density of 1 × 10^4^ cells per well in a 24-well plate, in which the top layer of agar was enhanced with 2X DMEM containing 0.3% Noble agar and 5% FBS, and the lower layer comprised 2X DMEM containing 0.5% agar and 5% FBS. Following solidification, culture medium (500 µL) was added and cultured for 3 weeks at 37 °C. The number of colonies larger than 50 µm was recorded. The SKOV3 cell line, which generated colonies within 15 days of implantation, was employed as a positive control.

### 4.12. Xenograft in Mice

Female athymic BLAB/c nu/nu and C.B-17/Icr-scid/scidJc1 mice (4 weeks old) were used in the mouse xenograft experiment (CLEA Japan, Inc., Shizuoka, Japan). Five mice were included in each experimental group for both nude and SCID mice. Subcutaneous and intraperitoneal injections of cultured cells (1 × 10^7^ cells/mL) were made on the left flank of mice. Over a few months or until the mice died, tumor growth was studied. Mice injected with immortalized HMOsisEC7 cells were monitored for 8–10 months alongside the other injected mice to confirm a non-transformed phenotype.

### 4.13. Statistical Analysis

Based on experiments conducted in triplicate, the data were reported as the mean ± SD. Statistical analyses were carried out using the Student’s *t*-test with the SPSS program (version 27, IBM, Armonk, NY, USA), and a *p*-value less than 0.05 was regarded as statistically significant.

## 5. Conclusions

We successfully created an in vitro and in vivo stepwise carcinogenesis model using immortalized endometriotic epithelial cells, in which introducing three specific genetic combinations (*ARID1A* KO/*KRAS* MT/overexpressed c-Myc or *ARID1A* KO/overexpressed AKT/overexpressed c-Myc) enabled the cells to form apparent tumors in immunocompromised mice, confirming that they are essential drivers for the development of ERONs. Of particular interest is the histological diversity depending upon the mouse species inoculated with the same genetic mutational cell line; OCCC phenotypes in SCID mice and OEC phenotypes in nude mice, suggesting the possible involvement of TIME (especially B-cell signaling) on histological outcomes. This model has the potential to facilitate future research on ERON development and the advancement of targeted therapies.

## Figures and Tables

**Table 1:** Xenograft results in SCID mice.

Mutant Type	Result (Mice with Tumor Formation/Inoculated Mice)	Remarks (Histology)
Wild type (HMOsisEC7)	0/5	
*ARID1A KO*/*PIK3CA* MT	0/5	
*ARID1A KO*/*KRAS* MT	0/5	
*ARID1A KO*/*KRAS* MT/AKT	2/5	No malignancy
*ARID1A KO*/*KRAS* MT/c-Myc	4/5	Clear cell carcinoma
*ARID1A KO*/AKT	0/5	
*ARID1A KO*/AKT/*KRAS*	2/5	No malignancy
*ARID1A KO*/AKT/c-Myc	3/5	Clear cell carcinoma

**Table 2:** Xenograft results in nude mice.

Mutant Type	Result (Mice with Tumor Formation/Inoculated Mice)	Remarks (Histology)
Wild type (HOMsisEC7)	0/5	
*ARID1A KO*/*PIK3CA* MT	0/5	
*ARID1A KO*/*KRAS* MT	0/5	
*ARID1A KO*/*KRAS* MT/AKT	2/5	No malignancy
*ARID1A KO*/*KRAS* MT/c-Myc	2/5	Endmetrioid carcinoma
*ARID1A KO*/AKT	0/5	
*ARID1A KO*/AKT/*KRAS*	2/5	No malignancy
*ARID1A KO*/AKT/c-Myc	1/5	No malignancy

## Data Availability

The data presented in this study are available on request from the corresponding authors.

## Supplementary Materials

The following supporting information can be downloaded at: https://www.mdpi.com/article/10.3390/ijms26051995/s1.

## Abbreviations

The following abbreviations are used in this manuscript:

ERON	Endometriosis-related ovarian neoplasm
OCCC	Ovarian clear cell carcinoma
OEC	Ovarian endometrioid carcinoma
KO	Knockout
KRAS	Kirsten rat sarcoma virus
ARID1A	AT-rich interaction domain 1A
PIK3CA	Phosphatidylinositol-4,5-bisphosphate 3-kinase catalytic subunit alpha
c-Myc	Cellular myelocytomatosis oncogene
AKT	Ak strain transforming
TIME	Tumor immune microenvironment
MT	Mutation
