# Supplementary material for: Establishment of a Novel In Vitro and In Vivo Model to Understand Molecular Carcinogenesis of Endometriosis-Related Ovarian Neoplasms
Source: Int J Mol Sci. 2025 Feb 25;26(5):1995. doi: 10.3390/ijms26051995 (PMC11901000; doi:10.3390/ijms26051995)
Supplement: Supplementary file 1 [file ijms-26-01995-s001.zip › ijms-3496796-supplementary.pdf]

Article

Establishment of a novel in vitro and in vivo model to understand molecular carcinogenesis of endometriosis-related ovarian neoplasms

Hasibul Islam Sohel[1], Tohru Kiyono[2], Umme Farzana Zahan[1], Sultana Razia[3], Masako Ishikawa[1], Hitomi Yamashita[1], Kosuke Kanno[1], Shahataj Begum Sonia[1], Kentaro Nakayama[4*] and Satoru Kyo[1*]

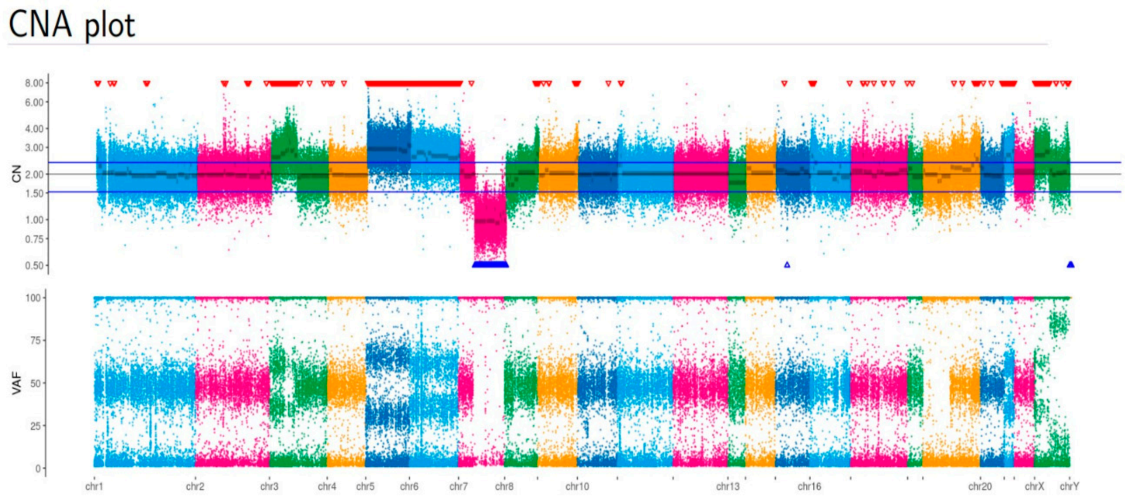

Supplementary Figure S1: Whole-exome sequencing reveals no significant alterations of representative driver candidates were detected throughout the entire chromosomes.

Supplementary Table S1. Whole exome sequencing result of HMOsisEC7.

Mutation type	Status
dMMR (Deficient Mismatch Repair)	No mutation
HRD (Homologous Recombinant deficiency)	No mutation
OG (Oncogene)	No mutation
TSG (Tumor Suppressor Gene)	No mutation
Other Gene Mutation	No mutation

Supplementary Table S2. Description of primary antibodies.

**Antibody Name**	**Manufacturer**	**Dilution Ratio**	MW (kDa)
ARID1A (PSG3)	Santa Cruz Biotechnology (sc-32761)	WB 1:500<br>IHC 1:100	165-320
Anti-phospho-p44/42 MAPK(Erk1/2) (Thr202/Tyr204)	Cell Signaling (4370)	WB 1:2000	42, 44
Anti-p44/42 MAPK (Erk1/2) (137F5)	Cell Signaling (4695)	WB 1:1000	42, 44
Anti-phospho-Akt (Ser473)	Cell Signaling (4060)	WB 1:2000	60
Anti-Akt (pan) (C67E7)	Cell Signaling (4691)	WB 1:1000	60
Anti-pan-Cytokeratin (C11)	Santa Cruz Biotechnology (sc-8018)	WB 1:1000<br>IHC 1:50	40-59
Anti-c-Myc	Cell Signaling (9402)	IHC 1:100	57-70
Anti-HNF-1β	Proteintech	IHC 1:200	58-60
GAPDH	Cell signaling (14C10)	1:1000 (WB)	37

Note: The aforementioned antibodies are utilized for western blot and immunohistochemistry analyses.

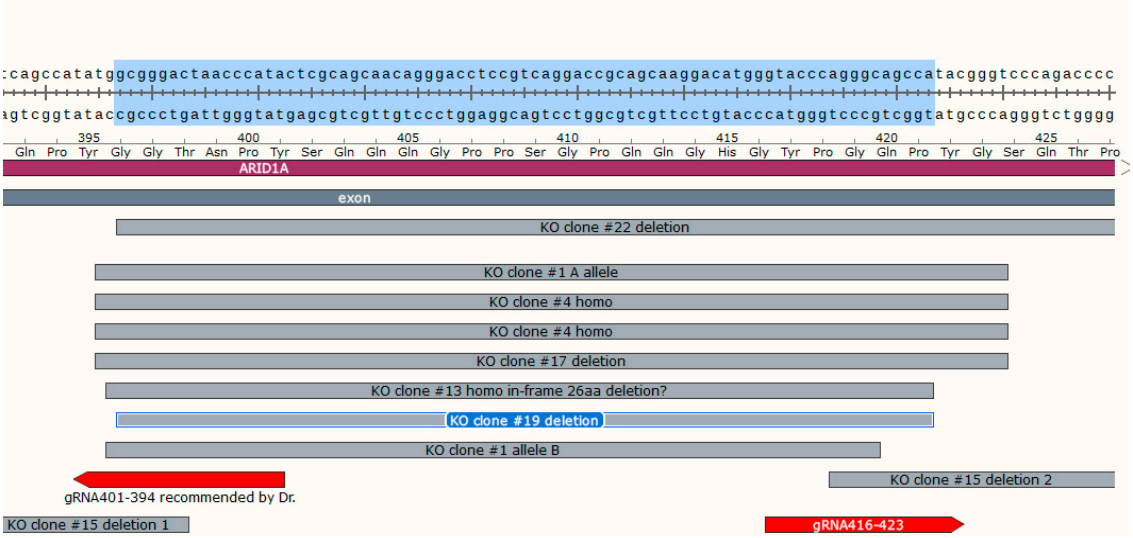

Supplementary Figure S2: *ARID1A* gene knockout using CRISPR CAS9.

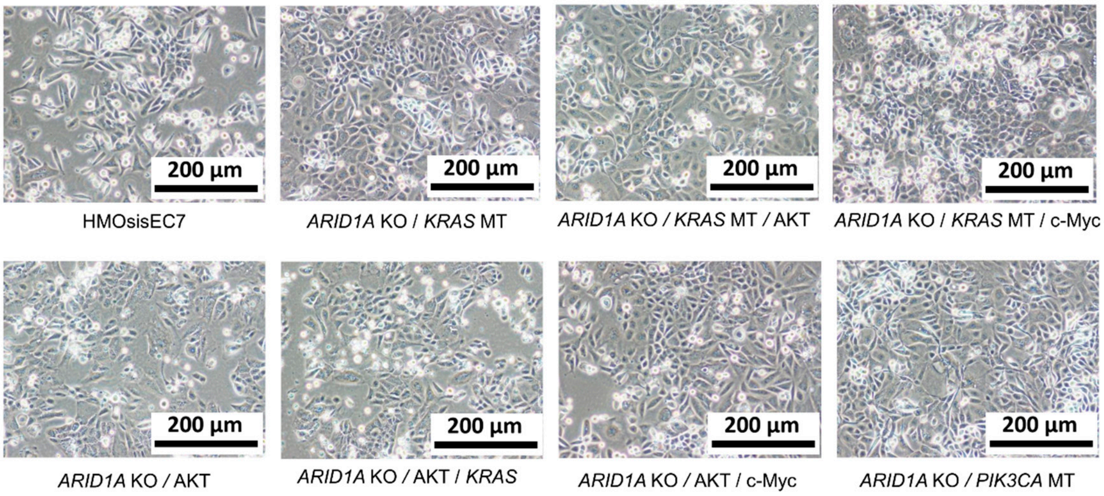

Supplementary Figure S3: Morphological characteristics of HMOsisEC7 cells with various genetic manipulations.

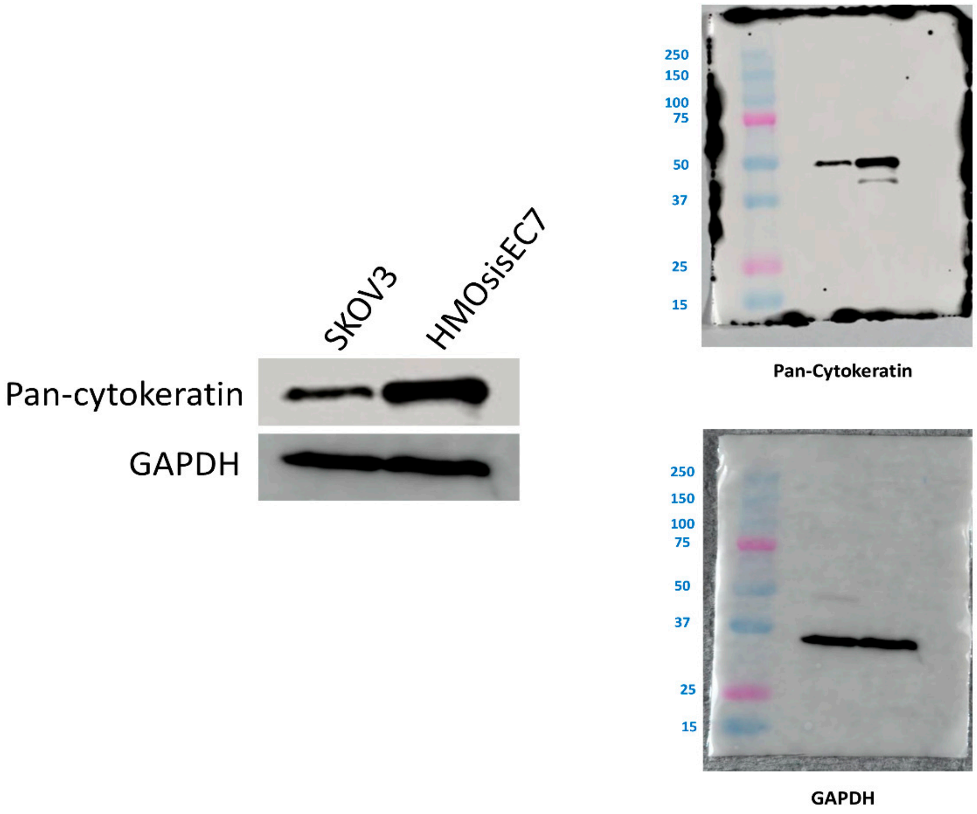

Supplementary Figure S4: Western blot analysis of pan-Cytokeratin in immortalized HMOsisEC7 cells.

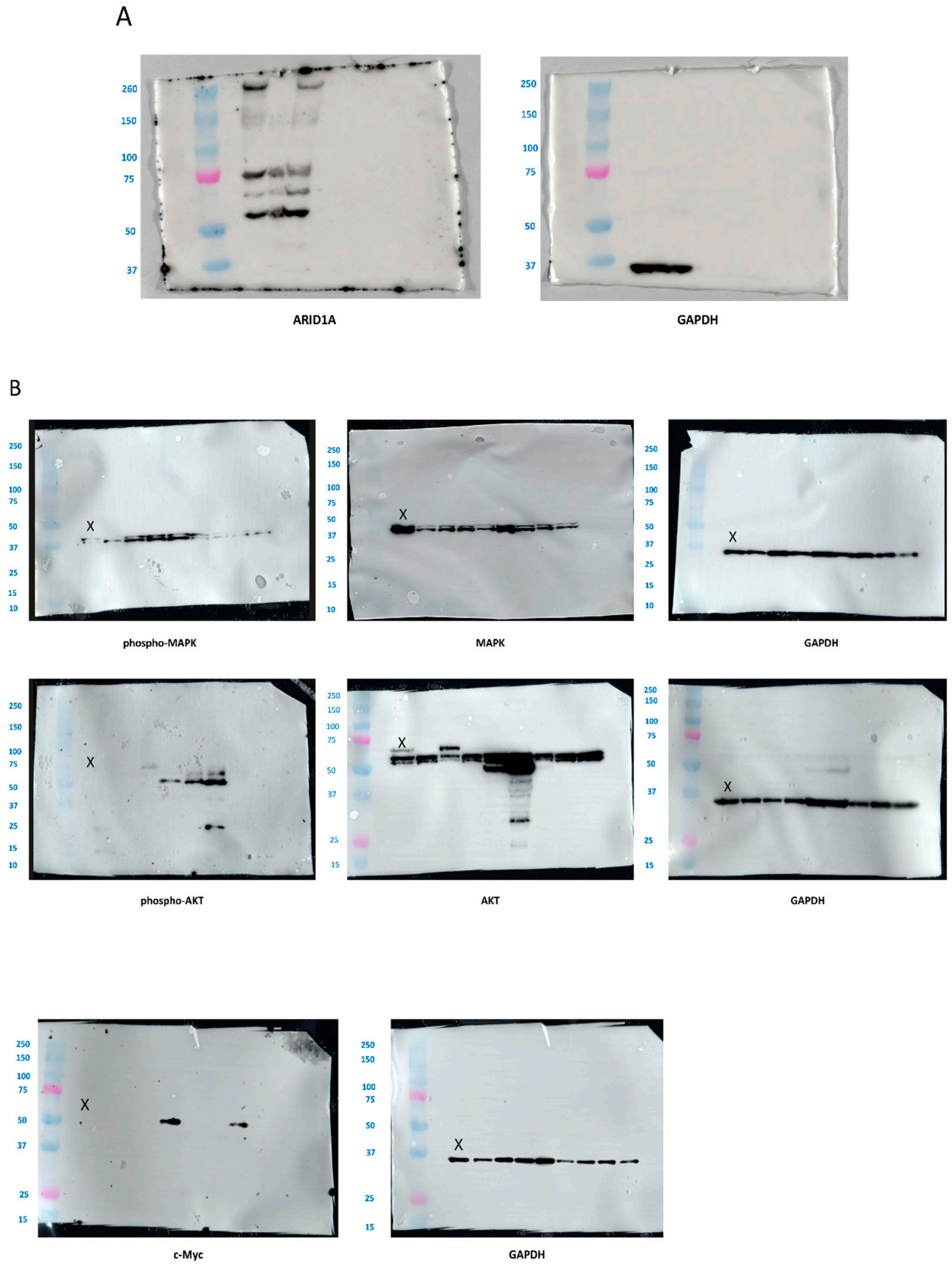

Supplementary Figure S5: Western blot analysis confirming the expression of ARID1A or other genes in HMOsisEC7 cells**.**

A: Expression of ARID1A in cells with *ARID1A* KO compared to parental HMOsisEC7 cells and the SKOV3 cell line.

B: Expression of various target genes in cells with overexpressing *KRAS* or

PIK3CA mutant alleles or overexpressing constitutively activated AKT or c-Myc. The names of each cell type and the various genetic manipulations are listed in the gel images. Lane marked X is not used in the main figure.

KO: knock out. MT: mutation. p: phosphorylated.

A

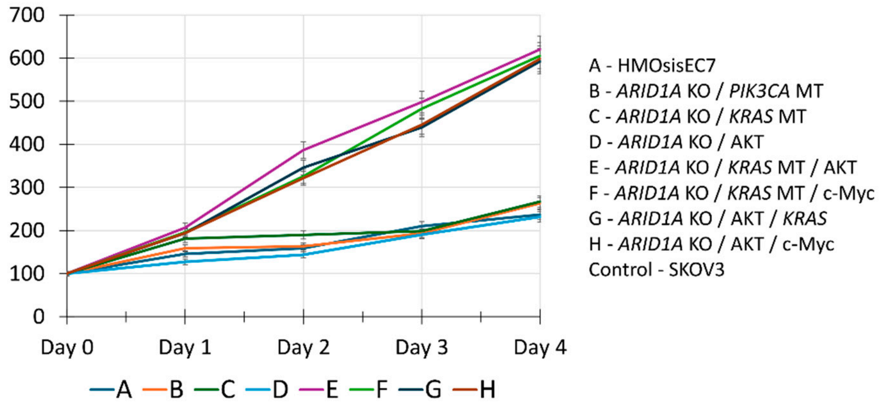

B

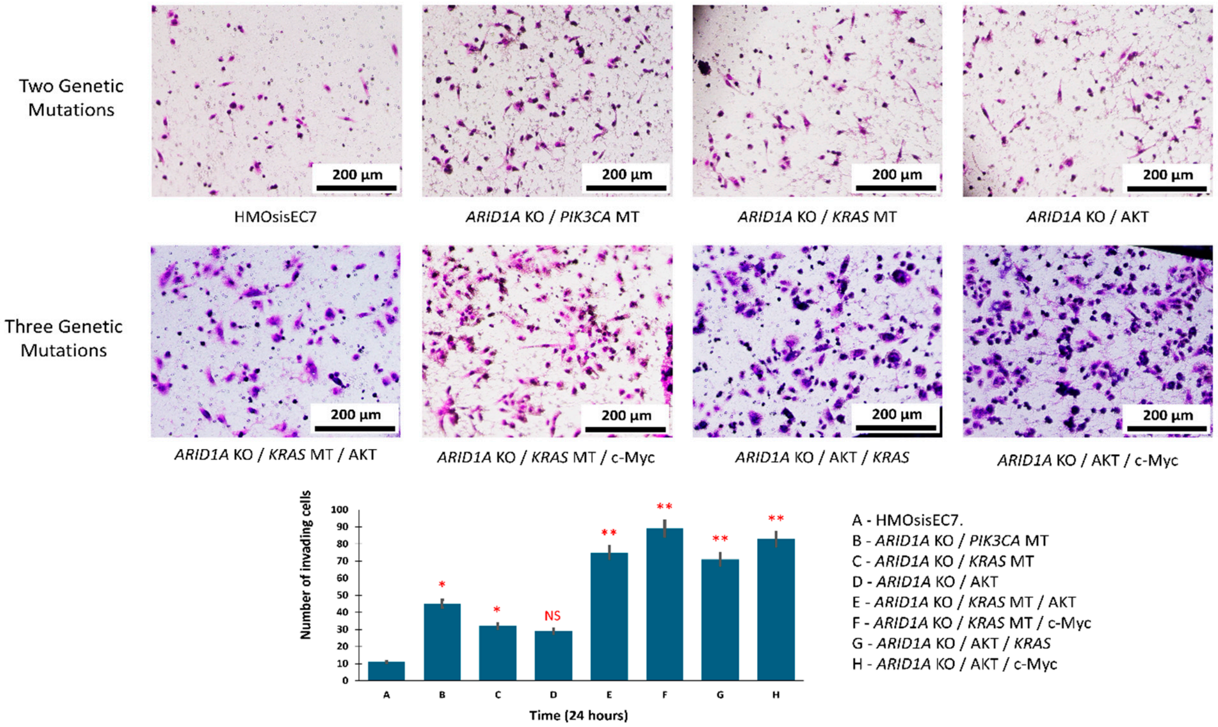

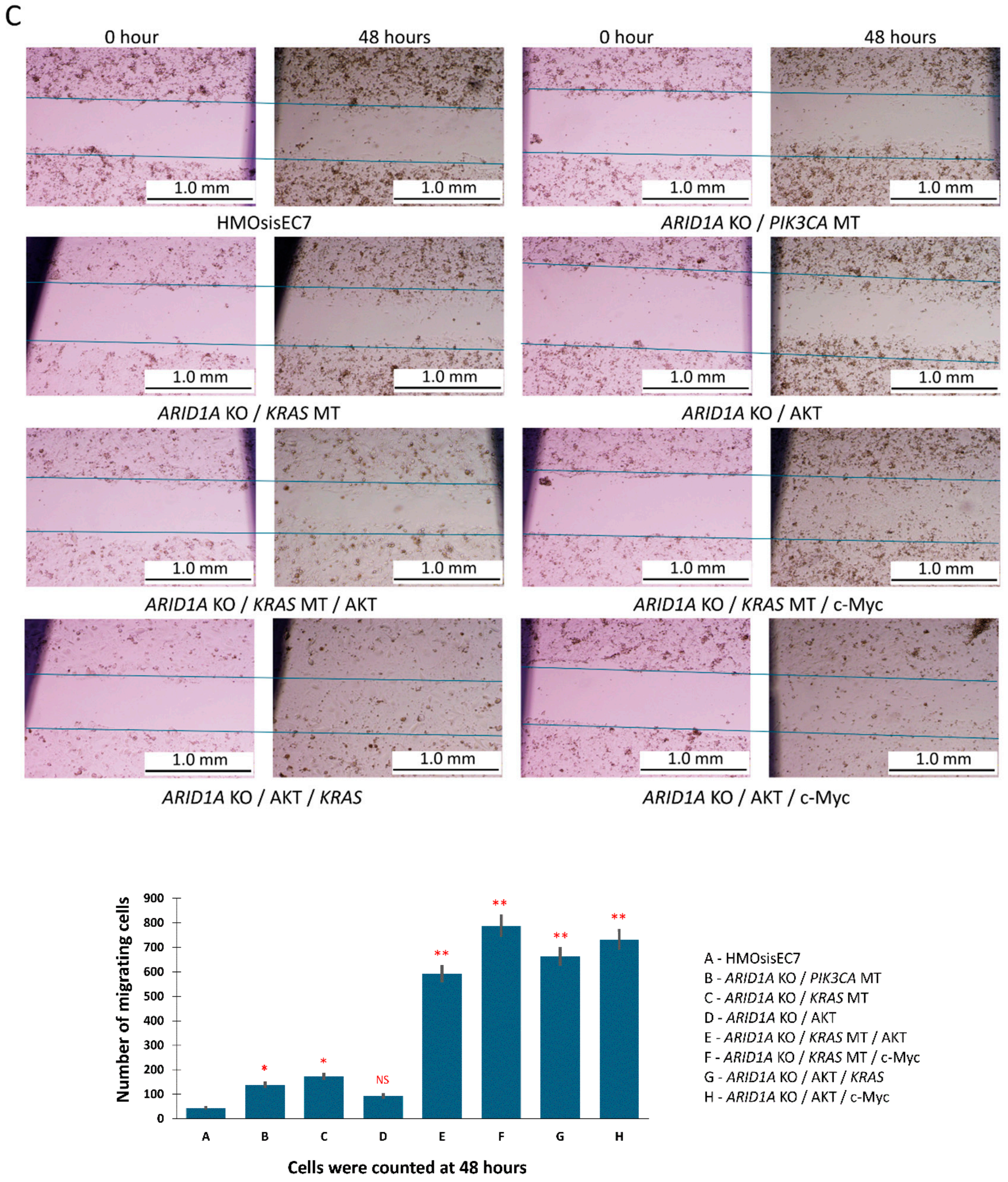

Supplementary Figure S6: Growth, wound healing, and invasion assays of HMOsisEC7 cells with various genetic manipulations.

A: MTT assay. The number of proliferating cells is shown for each cell type with various genetic manipulations on different days.

B: Invasion assay. The number of invading cells is shown in each cell type with various genetic manipulations. *$p < 0.05$, **$p < 0.01$.

C: Wound healing assay. The number of migrating cells is shown in each cell type with various genetic manipulations. *$p < 0.05$, **$p < 0.01$.

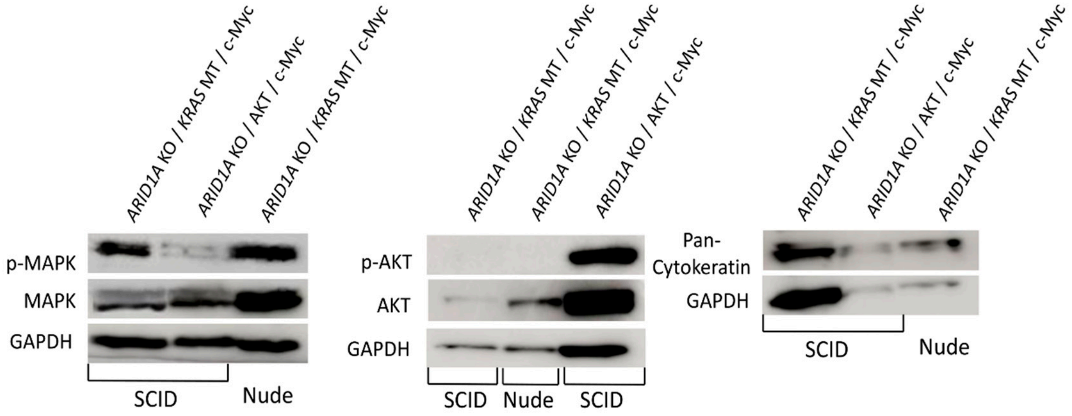
ARID1A KO / KRAS MT / c-Myc
ARID1A KO / AKT / c-Myc
ARID1A KO / KRAS MT / c-Myc
p-MAPK
MAPK
GAPDH
SCID
Nude
ARID1A KO / KRAS MT / c-Myc
ARID1A KO / KRAS MT / c-Myc
ARID1A KO / AKT / c-Myc
p-AKT
AKT
GAPDH
SCID
Nude
SCID
ARID1A KO / KRAS MT / c-Myc
ARID1A KO / AKT / c-Myc
ARID1A KO / KRAS MT / c-Myc
Pan-Cytokeratin
GAPDH
SCID
Nude

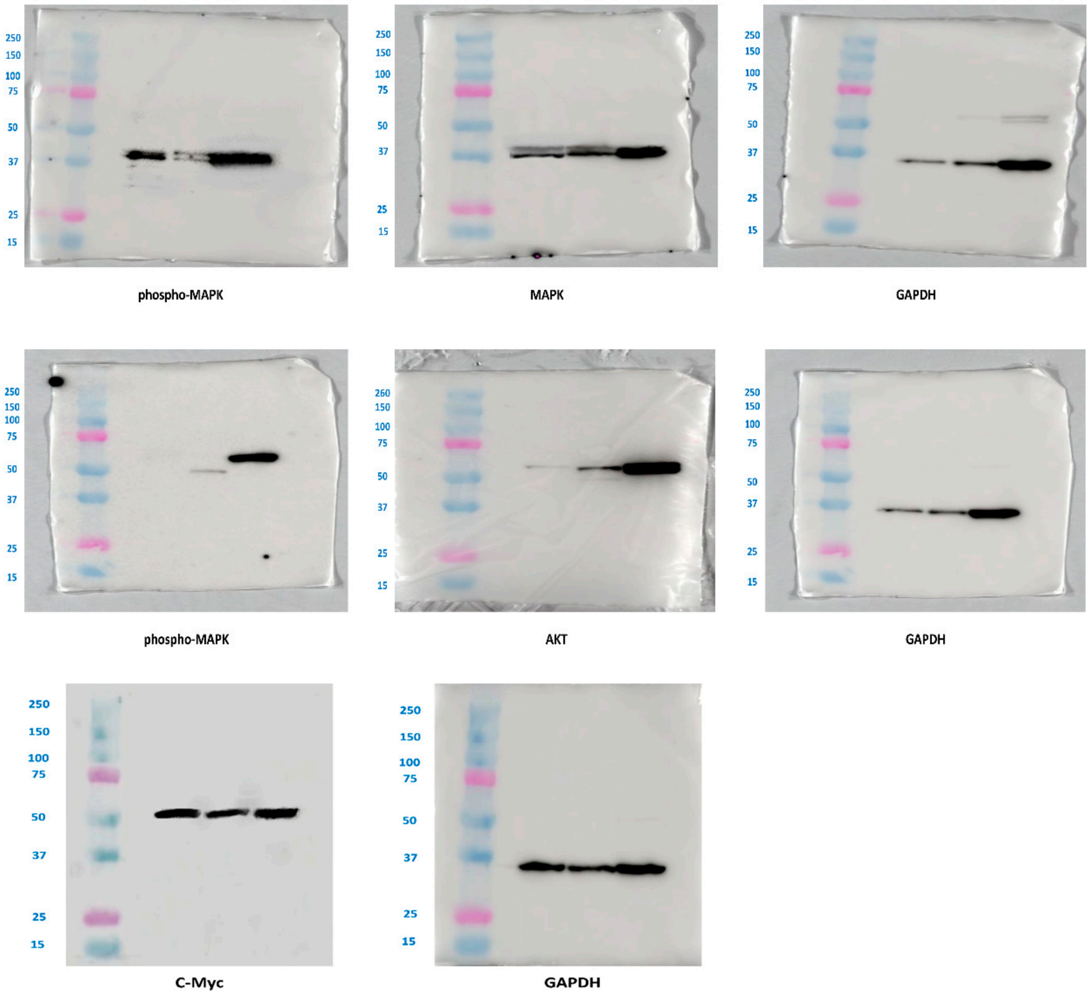
phospho-MAPK
MAPK
GAPDH
phospho-MAPK
AKT
GAPDH
C-Myc
GAPDH

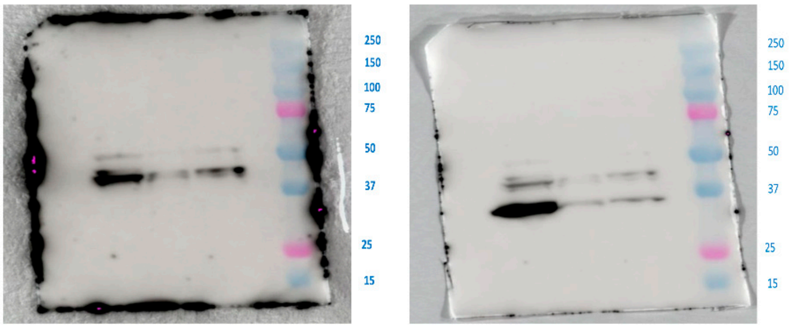

Pan-Cytokeratin GAPDH

Supplementary Figure S7: Analysis of xenograft tumors using Western blot. The expression levels of phospho-MAPK, pan-MAPK, phospho-AKT, pan-AKT and Pan-cytokeratin were assessed using Western blot analysis for SCID and Nude mouse tumors formed by each genetic combination. The positive expression of phospho-MAPK and phospho-AKT confirmed the activation of RAS/ERK and PI3K/AKT signaling pathways, and the positive expression of c-Myc showed its active role in these pathways. Positive expression of Pan-cytokeratin confirms epithelial origin. Tumor protein loading was assessed using a mouse monoclonal antibody against the housekeeping protein glyceraldehyde-3-phosphate dehydrogenase (GAPDH).
